# Trophoblast Enrichment by Maternal Immune-Cell Depletion Using CD45 and CD56 Surface Markers in Trophoblast Retrieval and Isolation from the Cervix (TRIC)

**DOI:** 10.3390/diagnostics16172714

**Published:** 2026-08-25

**Authors:** Heeyeon Jang, Hyun Ji Son, Minyeon Go, Jong Chul Kim, Ji Eun Park, Hyunjin Kim, Hee Jin Park, Soo Hyun Kim, Sung Shin Shim, You Jung Han, Young Jin Lee, Sung Han Shim, Dong Hyun Cha

**Affiliations:** 1 Department of Biomedical Science, College of Life Science, CHA University, Seongnam 13488, Republic of Korea; jhyeon@chabio.com (H.J.); raculase@chabio.com (M.G.); 2 Center for Genome Diagnostics, CHA Biotech Inc., Seoul 06125, Republic of Korea; hjson1030@chabio.com (H.J.S.); jckim43@chabio.com (J.C.K.); ditto6626@chabio.com (J.E.P.); hjkim0530@chabio.com (H.K.); younglee@chabio.com (Y.J.L.); 3 Department of Obstetrics and Gynecology, CHA Gangnam Medical Center, CHA University, Seoul 06125, Republic of Korea; coolsome72@chamc.co.kr (H.J.P.); soohyunkim@chamc.co.kr (S.H.K.); ogshinyss@chamc.co.kr (S.S.S.); hanyj1978@chamc.co.kr (Y.J.H.)

**Keywords:** trophoblast retrieval and isolation from the cervix (TRIC), prenatal testing, trophoblast, maternal immune cell

## Abstract

**Background**: Trophoblast retrieval and isolation from the cervix (TRIC) has emerged as a promising alternative to invasive prenatal diagnostic procedures. However, contamination by maternal immune cells remains a major challenge that may compromise trophoblast purity and the reliability of downstream fetal genetic analyses. **Methods**: Maternal immune cells were selectively depleted by immunomagnetic sorting using antibodies targeting CD45 or CD56. The remaining cells were subsequently enriched for HLA-G-positive trophoblasts and characterized by immunofluorescence and gene-expression analyses using CD45, CD56, HLA-G, cytokeratin 7 (CK7), and β-human chorionic gonadotropin (β-hCG). **Results**: Compared with CD45-mediated depletion, CD56-mediated depletion demonstrated more efficient removal of maternal immune cells, as indicated by significantly reduced CD56 expression. CK7 expression showed an increasing trend following CD56 depletion, whereas β-hCG expression remained largely unchanged. Immunofluorescence analysis further demonstrated a significant increase in the proportion of CK7^+^/β-hCG^+^ trophoblast cells after CD56 depletion. **Conclusions**: Among the evaluated depletion strategies, CD56-mediated depletion demonstrated a more favorable profile for trophoblast-associated characteristics than CD45-mediated depletion, suggesting its potential contribution to further methodological optimization of trophoblast isolation in TRIC-based noninvasive prenatal genetic testing.

## 1. Introduction

Trophoblast retrieval and isolation from the cervix (TRIC) is a noninvasive method for obtaining fetal trophoblast cells during early pregnancy, typically between 5 and 20 weeks of gestation. Using TRIC, fetal-derived trophoblast cells can be collected noninvasively from the endocervical canal, thereby minimizing procedural risks associated with invasive prenatal testing [1]. In contrast, noninvasive prenatal testing (NIPT), which analyzes cell-free DNA (cfDNA) circulating in maternal plasma, provides high sensitivity (98–99%) for detecting common chromosomal aneuploidies involving chromosomes 13, 18, 21, and the sex chromosomes [2,3]. However, the diagnostic performance of NIPT depends on an adequate fetal cfDNA fraction, which is typically required to be approximately 4% or higher. Moreover, NIPT provides limited information regarding the fetal genome and may require invasive confirmatory procedures in cases with abnormal or inconclusive results [4]. Invasive diagnostic procedures, including chorionic villus sampling (CVS) and amniocentesis, provide direct fetal genetic information but carry a potential risk of miscarriage estimated at approximately 0.1–2% [5,6].

Trophoblast cells originate from the trophectoderm layer of the blastocyst and initiate invasion of the maternal endometrium during early placental development. Distinct trophoblast subtypes regulate placental morphogenesis by maintaining placental structural integrity and facilitating vascular remodeling [7,8]. In addition to their essential biological functions, trophoblast-derived cells and cell-free materials can enter the maternal circulation or be detected in cervical secretions, providing a cellular and molecular basis for the development of noninvasive prenatal diagnostic approaches [9]. The invasion, differentiation, and survival of trophoblasts are regulated by the maternal immune microenvironment within the decidua. During early pregnancy, decidual immune populations, including uterine natural killer (uNK) cells and lymphocyte subsets, establish an immune-tolerant environment that supports placental development and early gestational maintenance [10,11].

Despite its diagnostic potential, TRIC remains limited by maternal-cell contamination within cervical samples [12]. Because fetal trophoblasts are recovered through the cervical canal using a noninvasive sampling approach, maternal cells are inevitably introduced during specimen collection. In particular, minor bleeding during cervical sampling may increase the contribution of maternal immune cells to the recovered cell population. Among these contaminating populations, immune cells expressing cluster of differentiation 45 (CD45) or cluster of differentiation 56 (CD56) represent major maternal cell components within cervical specimens.

CD45 is a pan-leukocyte marker expressed across hematopoietic immune cell populations, whereas CD56 is predominantly expressed by natural killer (NK) cells, which are abundant in maternal tissues during early pregnancy. Because maternal immune cells express distinct surface antigens, selective antibody-mediated depletion provides a strategy for reducing maternal-cell contamination. Therefore, we applied nanoparticle-based immunomagnetic separation targeting CD45 and CD56 to selectively remove maternal immune cells before Human leukocyte antigen G (HLA-G)-mediated trophoblast enrichment. This approach was designed to improve the recovery of fetal trophoblast populations with reduced maternal-cell interference, thereby enhancing the reliability of downstream molecular diagnostic analyses.

In this study, we aimed to improve the purity of fetal trophoblast cells isolated from cervical samples by evaluating immune cell depletion strategies targeting maternal-specific markers. We further developed a TRIC-based enrichment workflow to facilitate the application of fetal cell-based approaches for noninvasive prenatal genetic diagnosis.

## 2. Materials and Methods

### 2.1. Patient Characterization

Endocervical samples were obtained from pregnant women (*n* = 19) who visited Gangnam CHA Hospital between May 2020 and December 2023. All participants provided written informed consent prior to sample collection. The inclusion criteria were singleton pregnancies between 6 and 17 weeks of gestation. Pregnancies complicated by vanishing twins, miscarriage, or multiple gestations were excluded. For each participant, fetal sex, gestational age, and the total number of recovered endocervical cells following sampling and washing were recorded (Table 1). This study was approved by the Institutional Review Board of Gangnam CHA Medical Center (GCI 2020-10-007).

### 2.2. Endocervical Cell Sampling

Endocervical cells were collected using a cytobrush commonly used for Pap smear procedures. The cytobrush was inserted into the cervix, rotated 360°, and transferred into 10 mL of Hanks’ Balanced Salt Solution (HBSS; Gibco, Waltham, MA, USA) for transport. In the laboratory, cervical mucus was thoroughly dispersed in the medium to release cellular components. The cell suspension was centrifuged at 2000 rpm for 5 min, and the supernatant was removed. Residual mucus was removed by three sequential washes with 10 mL of cold Dulbecco’s phosphate-buffered saline (DPBS; Welgene, Gyeongsan, Republic of Korea). To generate a single-cell suspension, cells were treated with trypsin–ethylenediaminetetraacetic acid (trypsin–EDTA; Gibco, Waltham, MA, USA) for 3 min at room temperature (25 °C), followed by three additional washes with 1 mL DPBS. Cells were subsequently fixed with 4% paraformaldehyde (PFA; Bio-solution Co., Ltd., Suwon-si, Republic of Kore) for 10 min at 4 °C and washed three times with 1 mL DPBS to remove residual fixative. The fixed cells were immediately subjected to magnetic cell separation.

### 2.3. Immunomagnetic Isolation of Trophoblast Cells from Endocervical Samples

Maternal immune cells were depleted from endocervical samples using 250 nm magnetic nanoparticles (Clemente Associates, Prescott, AZ, USA) conjugated with goat anti-mouse IgG antibodies. Mouse anti-CD56 (NCAM1; 100 µg; Abcam, Cambridge, UK) and mouse anti-CD45 (100 µg; Abcam) antibodies were used as immune cell-specific markers (Table 2). For immunomagnetic depletion, 20 µL of magnetic nanoparticles was used for approximately 2.5 × 10^5^ endocervical cells as the target input for each reaction.

For antibody conjugation, each 100 µL reaction contained 20 µL magnetic nanoparticles and 2 µg of either anti-CD45 or anti-CD56 antibody in DPBS containing 1% bovine serum albumin (BSA; Bovogen Biologicals Pty Ltd, Melbourne, Australia). The mixture was vortexed and incubated overnight at 4 °C. Subsequently, approximately 2.5 × 10^5^ endocervical cells were incubated with antibody-conjugated nanoparticles overnight at 4 °C with gentle inversion. Following immune-cell depletion, the unbound cell fraction, which contained putative trophoblast cells, was subjected to HLA-G-based isolation using anti-HLA-G antibody (clone 4H84; 10 µg/mL; BD Biosciences, San Jose, CA, USA)-conjugated nanoparticles. HLA-G-positive cells bound to the magnetic nanoparticles were isolated using magnetic separation and defined as the enriched trophoblast population, whereas the unbound fraction was considered the residual maternal cell population. Approximately 2.4 × 10^5^ endocervical cells, corresponding to the mean input cell number across samples, were used as the standardized reference for normalization of HLA-G-positive cell proportions. Because the number of recovered endocervical cells varied among individual specimens, all specimens could not be included in all experimental groups (NC, CD45 depletion, and CD56 depletion). To maintain a standardized input cell number for each experimental condition, specimens were allocated according to their available cell yield. Consequently, the number of specimens included in each experimental group differed among analyses.

### 2.4. Real-Time PCR

RNA was extracted from isolated trophoblast and immune cell populations using the RNeasy Mini Kit (QIAGEN, Hilden, Germany) according to the manufacturer’s instructions. A minimum of 1000 cells was used for each RNA extraction. RNA concentration was measured using a NanoDrop™ 2000 spectrophotometer (Thermo Scientific, Waltham, MA, USA). Complementary DNA (cDNA) was synthesized using the SuperScript™ III First-Strand Synthesis System (Invitrogen, Carlsbad, CA, USA). Quantitative PCR was performed using gene-specific primers obtained from Bioneer Co., Ltd. (Daejeon, Republic of Korea) and the FastStart SYBR Green Master Kit (Roche, Basel, Switzerland) (Table 3). Amplification was performed using the following cycling conditions: 95 °C for 10 min, followed by 39 cycles of 95 °C for 15 s, 60 °C for 1 min, and 72 °C for 1 min. Reactions were conducted using a CFX PCR Detection System (Bio-Rad, Hercules, CA, USA). β-actin was used as the internal reference gene. Each biological specimen was analyzed in technical triplicate, and the mean Ct value of the three technical replicates was used as a single observation for each specimen. Statistical analyses were performed using the biological specimens as independent experimental units. Results are presented as mean ± SE based on the independent biological specimens.

### 2.5. Immunofluorescence

HLA-G-positive cells isolated following CD45 or CD56 selection were cytocentrifuged onto slides using the Cytospin 7620 (Wescor Inc., Logan, UT, USA). A total of 100 HLA-G-positive cells were deposited onto each slide at 1500 rpm for 5 min in 200 µL DPBS. Cells were blocked with DPBS containing 3% BSA for 1 h at room temperature. Primary antibodies against β-human chorionic gonadotropin (β-hCG; 10 µg/mL; 5H4-E2, Thermo Scientific), CD45, and CD56 were diluted in PBS containing 1% BSA and incubated overnight at 4 °C. After three washes with DPBS, anti-Cytokeratin 7 (CK7; 10 µg/mL; ab181598, abcam) antibody was applied in PBS containing 1% BSA for 3 h at room temperature. Following additional DPBS washes, Alexa Fluor^®^ 555-conjugated goat anti-mouse IgG secondary antibody (5 µg/mL; Invitrogen) and Alexa Fluor^®^ 488-conjugated goat anti-rabbit IgG antibody (5 µg/mL; Invitrogen) were applied for 1 h in the dark at room temperature. Nuclear staining was performed with 4′,6-diamidino-2-phenylindole (DAPI; 1 µg/mL; Invitrogen) for 10 min. Slides were subsequently washed and mounted using mounting medium (Vector Laboratories, Burlingame, CA, USA). Fluorescence images were acquired using an Axio Imager 2 microscope (Carl Zeiss, Thornwood, NY, USA). The proportions of marker-positive cells were determined by counting stained cells relative to the total number of cells analyzed.

### 2.6. Statistical Analysis

Statistical analyses were performed using R software (version 4.0.2). Data are presented as the mean ± standard deviation (SD) unless otherwise indicated. Because the NC, CD45-depleted, and CD56-depleted conditions were generated from the same cervical specimens, comparisons among the three matched conditions were performed using the Friedman test. Post hoc pairwise comparisons were performed using the Wilcoxon signed-rank test with Holm adjustment for multiple comparisons. Only specimens with complete observations across all three conditions were included in the three-group analysis (*n* = 16). Pairwise comparisons were likewise performed using only matched specimens with observations available under both conditions being compared. Statistical significance was defined as an adjusted *p* value < 0.05.

## 3. Results

### 3.1. Comparison of CD45- and CD56-Based Immune Cell Depletion

**Figure 1:**
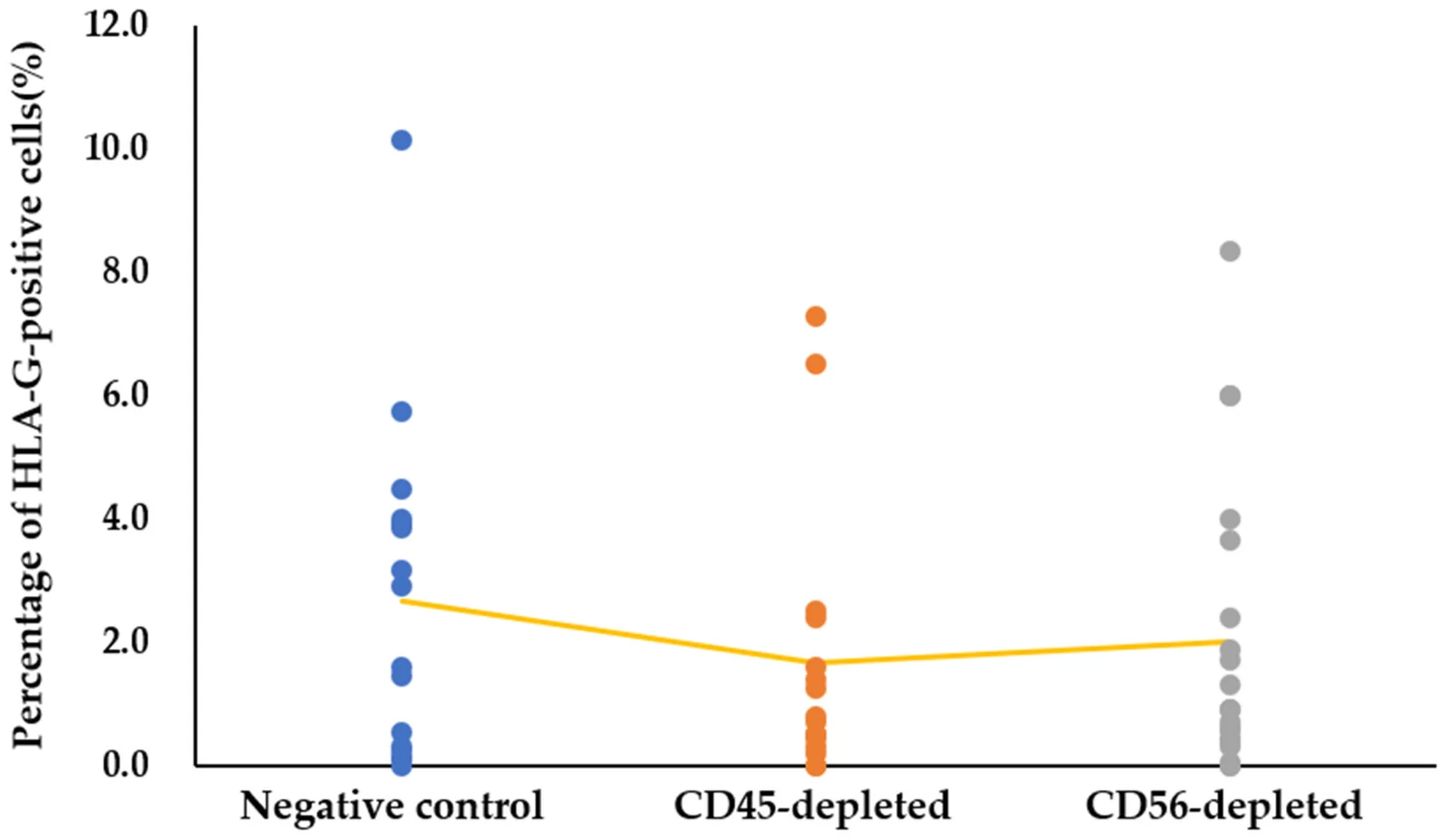
Comparison of HLA-G-positive cell recovery following CD45-depleted or CD56-depleted. Endocervical cells were subjected to immune cell depletion using anti-CD45- or anti-CD56-conjugated magnetic nanoparticles. HLA-G-positive trophoblasts were subsequently isolated and counted. The proportion of HLA-G-positive cells was calculated relative to the total number of input endocervical cells used for separation (approximately 2.4 × 10^5^ cells). The NC group underwent HLA-G isolation without prior immune cell depletion. Data are presented as individual paired samples with mean ± SD. Statistical significance was assessed using the Friedman test, followed by post hoc pairwise comparisons using the Wilcoxon signed-rank test with Holm adjustment for multiple comparisons.

The total number of cells and HLA-G-positive cells were counted to determine the proportion of HLA-G-positive cells before and after immune cell depletion. The proportion of HLA-G-positive cells was calculated by dividing the number of HLA-G-positive cells by the total number of input cells used for separation (approximately 2.4 × 10^5^ cells). Because the denominator included the entire input cell population, the calculated percentages were low. These values represent the relative abundance of HLA-G-positive cells among the cells subjected to separation and do not represent the absolute proportion of fetal trophoblast cells within the original cervical sample. The proportions of HLA-G-positive cells were compared among the non-depleted control (NC), CD45-depleted, and CD56-depleted groups (Figure 1, Supplementary Table S1). Both CD45- and CD56-mediated depletion resulted in a reduction in total recovered cell numbers. Although a decreasing trend in the proportion of HLA-G-positive cells was observed following both depletion strategies, no statistically significant differences were identified.

To further evaluate changes in cell recovery following immune cell depletion, the total number of recovered endocervical cells and HLA-G-positive cells was analyzed for each group (Table 4). No significant differences were observed in the number of recovered endocervical cells among the three groups after cervical fluid washing. Approximately 2.5 × 10^5^ endocervical cells per sample were used as the target input for magnetic-activated cell sorting (MACS); however, the actual number varied according to the number of cells recovered from each participant. The NC group was included as a reference group without immune cell depletion, whereas the CD45-depleted and CD56-depleted groups underwent immune cell depletion using anti-CD45 and anti-CD56 antibodies, respectively. Following immune cell depletion, HLA-G-positive cells were isolated using anti-HLA-G antibodies. No significant differences in HLA-G-positive cell counts were observed among the three groups. Although similar numbers of input cells were used for each sample, the number of recovered cells showed a decreasing tendency following immune cell depletion and subsequent HLA-G-positive cell isolation.

**Figure 2:**
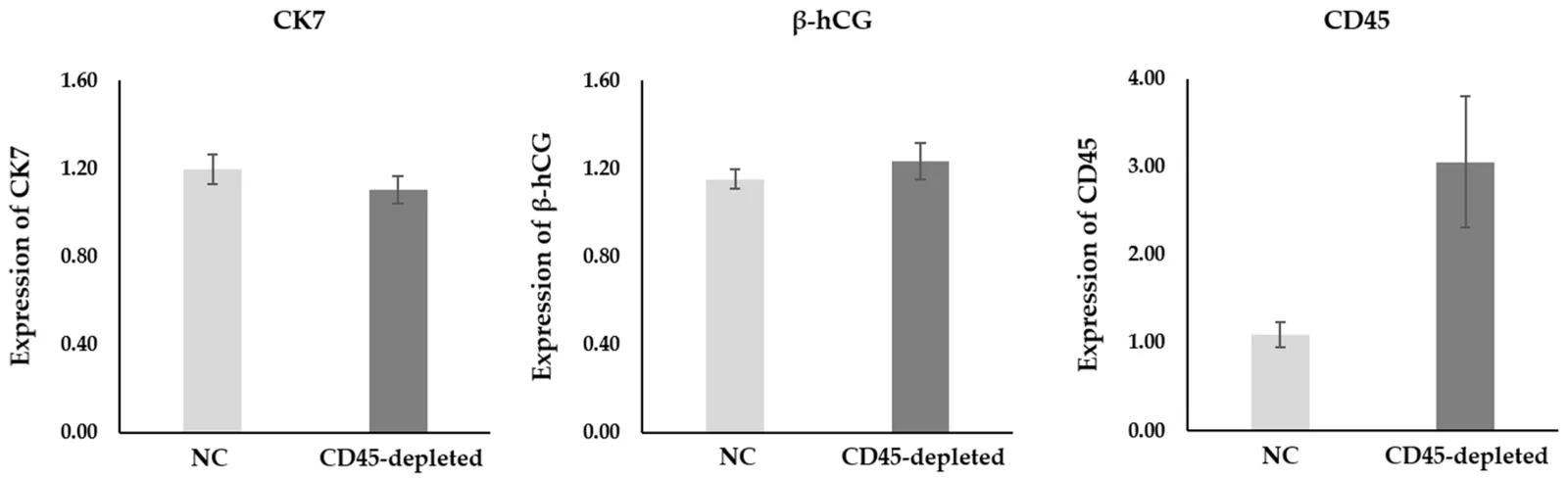
Gene-expression analysis of HLA-G-positive trophoblasts following CD45-mediated immune cell depletion. NC: non-depleted CD45 group. The CD45-depleted group underwent CD45-mediated immune cell depletion, whereas the NC group did not undergo CD45 depletion. HLA-G-positive trophoblasts were subsequently isolated, and the expression of CK7, β-hCG, and CD45 was analyzed by quantitative PCR. Each biological specimen was analyzed in technical triplicate, with the mean Ct value used for statistical analysis. Data are presented as individual paired samples with mean ± SD. Statistical significance was assessed using the Wilcoxon signed-rank test.

Gene expression analysis was performed using HLA-G-positive cells isolated from CD45-depleted and NC (non-depleted) groups, with nine independent biological specimens included in each group (*n* = 9). Compared with the NC group, the CD45-depleted group exhibited a tendency toward reduced CK7 expression and increased β-hCG expression; however, neither difference reached statistical significance. CD45 expression was also lower in the CD45-depleted group than in the NC group, although this difference was not statistically significant (Figure 2).

**Figure 3:**
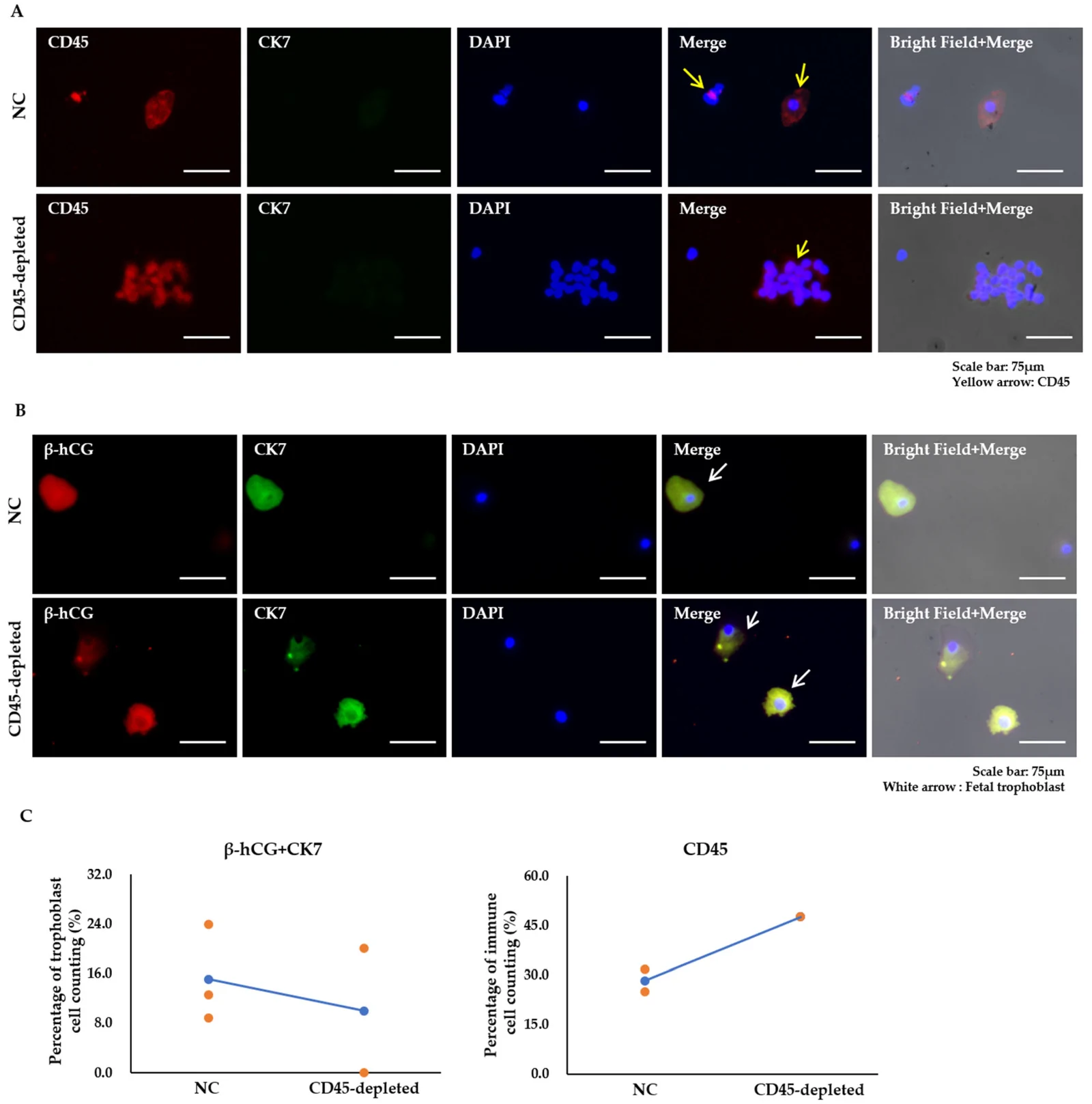
Immunofluorescence analysis of maternal immune cells and trophoblast markers following CD45-mediated immune cell depletion. NC: non-depleted CD45 group. HLA-G-positive cells were isolated from samples with CD45-mediated immune cell depletion (CD45-depleted) or without CD45-mediated immune cell depletion (NC). (**A**) Cells were stained for CD45 (red, RFP), CK7 (green, GFP), and DAPI (blue; nuclei). No CK7-positive cells were observed in the field; therefore, no green fluorescence signal is visible. Yellow arrows indicate CD45^+^ maternal immune cells. (**B**) Cells were stained for β-hCG (red, RFP), CK7 (green, GFP), and DAPI (blue; nuclei). White arrows indicate CK7^+^/β-hCG^+^ trophoblast cells. Images were acquired at ×200 magnification. (scale bar = 75 μm) (**C**) Quantitative analysis was performed by counting all cells across the entire slide and calculating the proportions of CK7^+^/β-hCG^+^ trophoblast cells and CD45-positive immune cells within the isolated HLA-G-positive cell fraction. Orange dots represent individual paired samples, and blue bars represent the mean ± SD.

Immunofluorescence analysis was performed using dual staining for CD45 and CK7 or CK7 and β-hCG to distinguish maternal immune cells (CD45^+^) from fetal trophoblast cells (CK7^+^/β-hCG^+^). CD45 staining exhibited a diffuse distribution before depletion and appeared as aggregated clusters after depletion. The absence of CD45 and CK7 co-localization indicated that CD45-positive cells represented maternal immune cells rather than fetal trophoblast cells (Figure 3A). No significant differences in CK7 and β-hCG staining patterns were observed among groups (Figure 3B). Quantitative analysis was performed by counting all cells present on each slide and determining the number of positively stained cells. The proportion of marker-positive cells was calculated based on the total number of counted cells. Following CD45 depletion, the proportion of CK7^+^/β-hCG^+^ cells showed a decreasing tendency, whereas CD45^+^ cells showed an increasing tendency; however, these differences were not statistically significant (Figure 3C).

**Figure 4:**
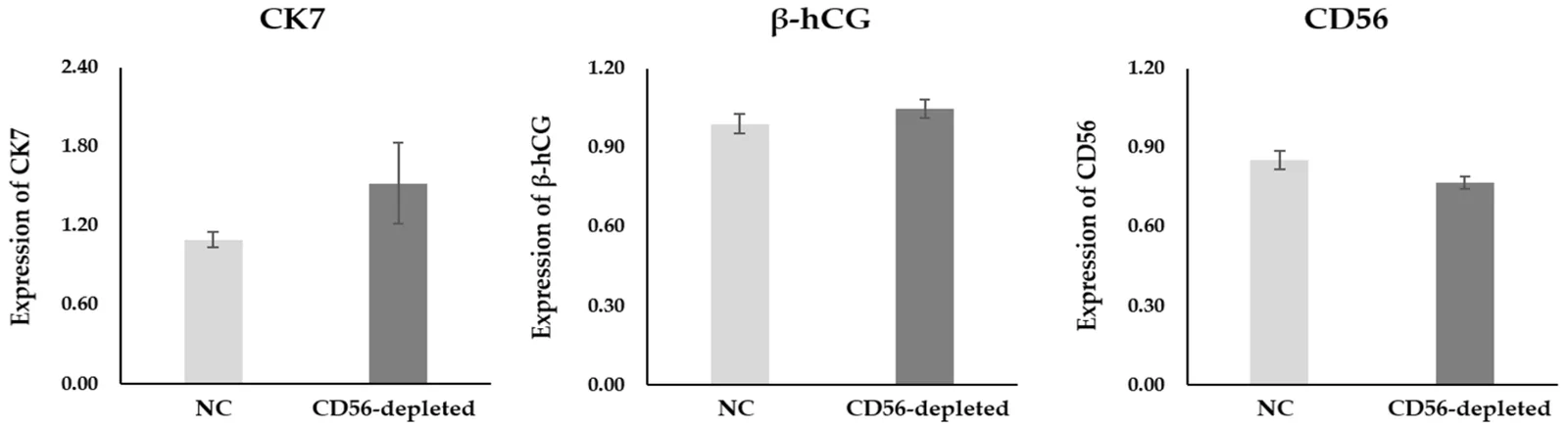
Gene-expression analysis of HLA-G-positive trophoblasts following CD56-mediated immune cell depletion. NC: non-depleted CD56 group. The CD56-depleted group underwent CD56-mediated immune cell depletion, whereas the NC group did not undergo CD56 depletion. HLA-G-positive trophoblasts were subsequently isolated, and the expression of CK7, β-hCG, and CD56 was analyzed by quantitative PCR. Each biological specimen was analyzed in technical triplicate, with the mean Ct value used for statistical analysis. Data are presented as individual paired samples with mean ± SD. Statistical significance was assessed using the Wilcoxon signed-rank test.

Gene expression analysis was performed using HLA-G-positive cells isolated from CD56-depleted and NC (non-depleted) groups, with nine independent biological specimens included in each group (*n* = 10). Compared with the NC group, the CD56-depleted group showed a tendency toward increased CK7 and β-hCG expression; however, these differences were not statistically significant. CD56 expression was lower in the CD56-depleted group than in the NC group, but this difference was also not statistically significant (Figure 4).

**Figure 5:**
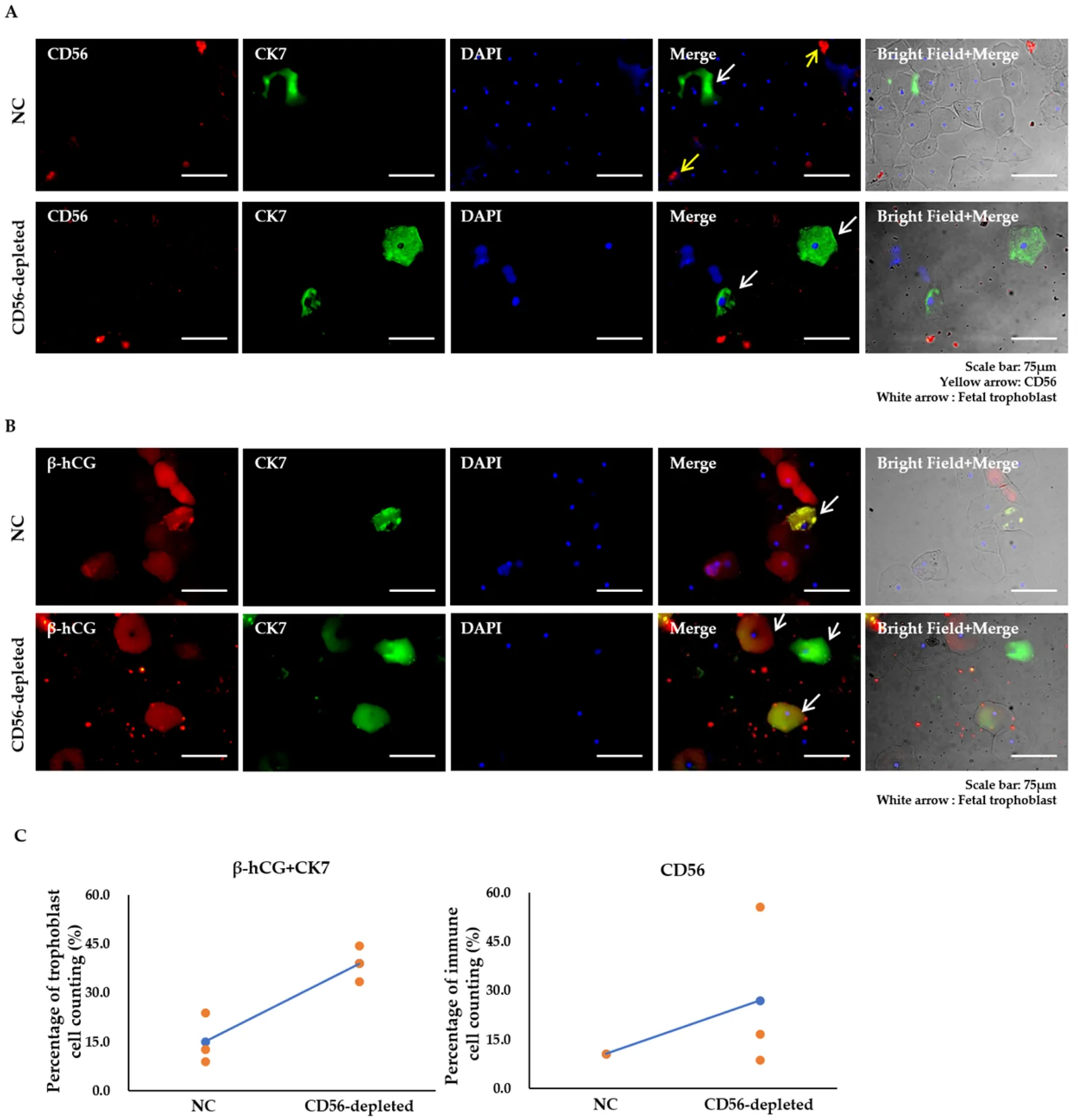
Immunofluorescence analysis of maternal immune cells and trophoblast markers following CD56-mediated immune cell depletion. NC: non-depleted CD56 group. HLA-G-positive cells were isolated from samples with CD56-mediated immune cell depletion (CD56-depleted) or without CD56-mediated immune cell depletion (NC). (**A**) Cells were stained for CD56 (red, RFP), CK7 (green, GFP), and DAPI (blue; nuclei). Yellow arrows indicate CD56^+^ maternal immune cells. (**B**) Cells were stained for β-hCG (red, RFP), CK7 (green, GFP), and DAPI (blue; nuclei). White arrows indicate CK7^+^/β-hCG^+^ trophoblast cells. Images were acquired at ×200 magnification. (scale bar = 75 μm) (**C**) Quantitative analysis was performed by counting all cells across the entire slide and calculating the proportions of CK7^+^/β-hCG^+^ trophoblast cells and CD56-positive immune cells within the isolated HLA-G-positive cell fraction. Orange dots represent individual paired samples, and blue bars represent the mean ± SD.

Immunofluorescence analysis was performed using dual staining for CD56 and CK7 or CK7 and β-hCG to distinguish maternal immune cells (CD56^+^) from fetal trophoblast cells (CK7^+^/β-hCG^+^). Representative images demonstrated a reduced number of cells following CD56 depletion (Figure 5A). Furthermore, CK7 and β-hCG dual staining revealed an increased number of CK7^+^/β-hCG^+^ cells in the CD56-depleted group compared with the non-depleted group (Figure 5B). Quantitative analysis was performed by counting all cells present on each slide and calculating the proportion of positively stained cells. The proportion of CK7^+^/β-hCG^+^ cells increased following CD56 depletion, although the difference was not statistically significant. Although representative images suggested reduced CD56-positive cells after depletion, quantitative analysis demonstrated a tendency toward an increased proportion of CD56-positive cells; however, this difference was not statistically significant (Figure 5C).

### 3.2. Comparative Evaluation of Depletion Strategies

**Figure 6:**
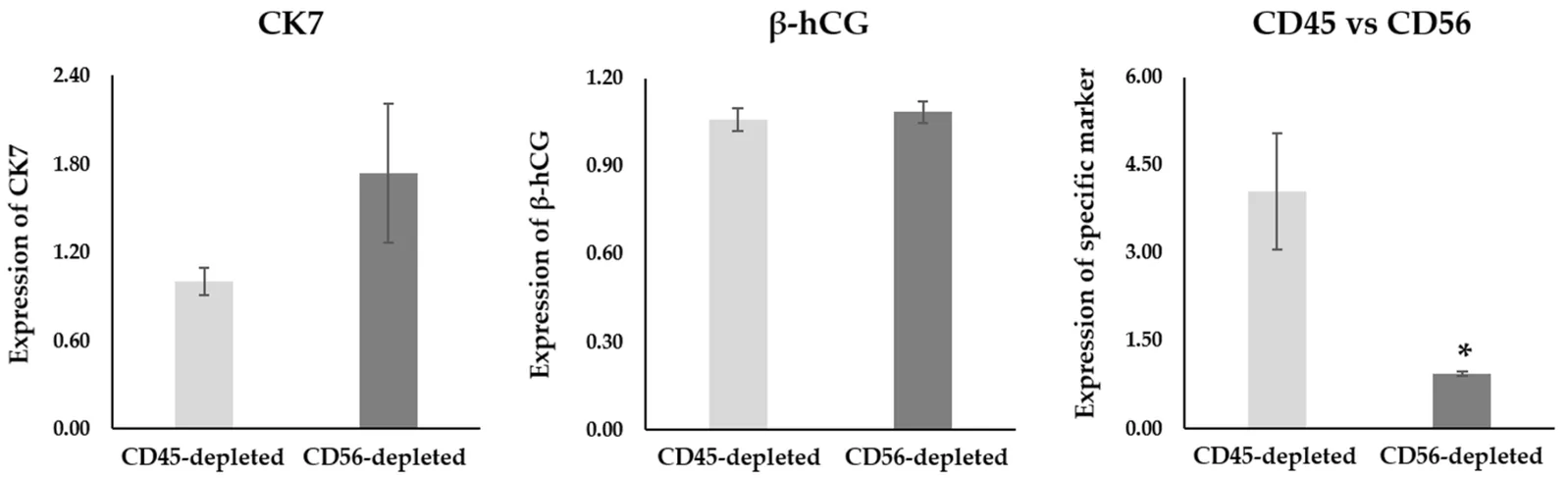
Comparison of gene-expression profiles following CD45- and CD56-mediated immune cell depletion. HLA-G-positive trophoblasts were isolated after CD45- or CD56-mediated immune cell depletion. Expression levels of CK7, β-hCG, CD45, and CD56 were compared to evaluate the effects of the two immune cell depletion strategies on trophoblast-associated and maternal immune-cell marker expression. Data are presented as individual paired samples with mean ± SD. Statistical significance was assessed using the Wilcoxon signed-rank test (*: *p* < 0.05).

Gene expression profiling demonstrated distinct effects between CD45- and CD56-mediated immune cell depletion strategies (Figure 6). Compared with CD45-mediated depletion, CD56-mediated depletion showed a tendency toward increased CK7 expression, whereas β-hCG expression remained comparable between the two groups. In contrast, CD56 expression was significantly reduced following CD56-mediated depletion compared with CD45-mediated depletion (*p* < 0.05), indicating more effective removal of CD56-positive maternal immune cells. These findings suggest that CD56-mediated depletion provides a more favorable approach for enriching trophoblast-associated cell populations compared with CD45-mediated depletion.

## 4. Discussion

Trophoblasts are differentiated cells derived from the trophectoderm surrounding the embryo at the blastocyst stage. Because trophoblasts are of fetal origin and contain fetal genetic information, they represent a potential target for noninvasive prenatal genetic analysis. During implantation, trophoblasts invade and migrate through the endometrium, contributing to placental formation and embryonic development. Trophoblasts differentiate into three major subtypes: cytotrophoblasts (CTBs), syncytiotrophoblasts (STBs), and extravillous trophoblasts (EVTs) [13]. Between 5 and 12 weeks of gestation, invasive EVTs anchor the developing villi and participate in endovascular migration. Previous studies have proposed two potential mechanisms explaining the presence of EVTs in cervical fluid. First, EVTs located at the placental margin may invade the uterine epithelium and enter the uterine cavity. Alternatively, EVTs may detach from the placental margin and be released directly into the uterine cavity. These cells may subsequently be transported through uterine and endometrial glandular secretions toward the endocervical canal, allowing for their recovery from cervical fluid [5]. However, cervical fluid contains both fetal trophoblasts and maternal immune cells, including NK cells and other leukocytes. Therefore, successful recovery of fetal cells from cervical samples requires strategies that minimize maternal-cell contamination during fetal genetic analysis [14].

In this study, we isolated and characterized placenta-derived trophoblast populations from cervical specimens by introducing maternal immune-cell depletion using CD45^+^ and CD56^+^ markers prior to HLA-G-positive trophoblast enrichment and gene-expression analysis. Previous TRIC optimization studies have also emphasized the need to minimize maternal-cell contamination to improve trophoblast retrieval, highlighting the importance of balancing maternal-cell depletion with trophoblast recovery during protocol optimization [15,16]. Therefore, we incorporated an additional depletion step to evaluate whether selective removal of maternal immune components could improve trophoblast enrichment.

Cervical fluid collected using a cytobrush is susceptible to maternal-cell admixture during sampling, which may interfere with fetal cell-based molecular analyses [17]. Minor bleeding during cervical sampling can introduce maternal blood-derived immune cells into collected specimens, representing a potential challenge for diagnostic processing. These limitations highlight the importance of optimizing sample preparation strategies to reduce maternal-cell interference in cervical-based prenatal diagnostic approaches. Similar observations have been reported in cytological applications, where factors affecting sample adequacy may influence diagnostic performance [18].

To reduce maternal-cell contamination, we applied CD45- and CD56-targeted immunomagnetic depletion and evaluated changes in trophoblast-associated characteristics. Although differences in the proportion of HLA-G-positive cells were not statistically significant among groups, maternal immune-cell marker expression demonstrated differential responses depending on the depletion strategy. These findings were further supported by fetal trophoblast-associated marker analysis, including gene-expression and immunofluorescence assessments.

Trophoblast identity was evaluated using previously established lineage-specific markers. Placental trophoblast subtypes exhibit distinct expression patterns; β-hCG and chorionic somatomammotropin hormone 1 are associated with STB characteristics, HLA-G is a representative marker of EVT, and CK7 is commonly used as a pan-trophoblast marker [9,19]. Li et al. isolated placental cell populations using MACS and confirmed the expression of HLA-G, CK7, β-hCG, CD45, and CD9, demonstrating the feasibility of marker-based trophoblast enrichment [20]. In addition, previous TRIC studies have identified placental-associated gene-expression profiles, including EVT-related transcripts, supporting the potential application of cervical trophoblasts for fetal genetic analysis [16,21].

In the CD45 depletion experiment, fetal trophoblast-associated markers were not increased following CD45-mediated depletion. Instead, CD45 expression remained elevated in the CD45-depleted group compared with the non-depleted group, as confirmed by qRT-PCR and immunofluorescence analyses. This finding suggests that CD45-based depletion may have limited efficiency in reducing maternal immune-cell contamination in cervical samples. One possible explanation is incomplete separation or cell aggregation during immunomagnetic sorting. Previous studies have reported that magnetic bead-based separation efficiency can be influenced by nonspecific interactions, bead–cell interactions, and bead-to-bead binding forces [22,23]. Such technical limitations may affect the recovery and characterization of target cell populations during immunomagnetic isolation. Consistent with these observations, increased cellular aggregation was observed following CD45 depletion (Figure 3A). Therefore, the observed increase in CD45 expression may be attributable, at least in part, to technical limitations of the depletion procedure rather than a true biological increase in maternal immune cells.

In contrast, CD56-mediated depletion demonstrated a more favorable profile for trophoblast-associated enrichment. CD56 expression was significantly reduced following CD56 depletion, and CK7 expression showed an increasing tendency, accompanied by an increased proportion of CK7^+^/β-hCG^+^ cells in immunofluorescence analysis. CD56 is highly expressed on NK cells, which are abundant in decidual tissues during early pregnancy. Decidual NK cells interact with EVTs and contribute to immune regulation and placental development [24]. Because cervical sampling may introduce maternal blood-derived CD56^+^ immune cells into specimens, selective depletion of CD56-positive cells may reduce maternal immune-cell interference during trophoblast isolation. Previous studies have successfully isolated maternal immune populations using CD56-based magnetic separation, supporting the utility of CD56 as a target marker for immune-cell depletion [25]. Alternative trophoblast enrichment strategies, including microfluidic isolation, flow cytometry, and single-cell technologies, have recently been explored for cell-based noninvasive prenatal diagnosis. Although these approaches enable advanced cellular characterization, they generally require specialized instrumentation and more complex analytical workflows. In contrast, the immunomagnetic separation strategy used in the present study may provide a useful approach for trophoblast isolation and enrichment prior to downstream molecular analyses.

The present findings indicate that CD56-mediated depletion provides a more favorable strategy than CD45-mediated depletion for enriching trophoblast-associated cell populations from cervical samples. However, this favorable profile primarily reflects changes in trophoblast-associated characteristics rather than a significant increase in the proportion of HLA-G-positive cells. Although immune-cell depletion may improve the relative representation of fetal trophoblast markers, the associated reduction in total cell recovery remains an important consideration for downstream molecular analyses. Despite this limitation, reducing maternal-cell contamination may increase the relative enrichment of fetal trophoblast cells, thereby providing a more suitable cell population for downstream molecular analyses, including single-cell sequencing. Therefore, optimization of cell recovery and enrichment balance will be important for further assessing the potential utility of TRIC-based fetal genetic testing. From a diagnostic perspective, increasing the proportion of fetal trophoblast-derived signals while minimizing maternal-cell interference may facilitate downstream molecular analyses, including chromosomal assessment and sequencing-based approaches. In addition, the relatively small sample sizes used for the gene-expression analyses (*n* = 9 per group for the CD45-depletion comparison and *n* = 10 per group for the CD56-depletion comparison) may have reduced the statistical power of the present study. Therefore, larger, adequately powered studies will be required to validate these findings and confirm their reproducibility. Trophoblast enrichment was assessed using established trophoblast-associated markers (HLA-G, CK7, and β-hCG) together with qRT-PCR and immunofluorescence analyses rather than fetal-specific genetic markers. Although these markers are widely used for trophoblast characterization, incorporation of fetal-specific genetic markers in future studies will provide more direct validation of trophoblast purity and maternal-cell depletion.

The present study did not evaluate fetal DNA recovery, sequencing performance, fetal fraction, or diagnostic accuracy; therefore, whether the observed changes in trophoblast-associated characteristics translate into enhanced clinical performance remains to be determined. Future studies involving larger cohorts, optimized enrichment strategies, and comprehensive genomic analyses will be required to determine its potential clinical utility. Taken together, these findings provide a foundation for further methodological development and optimization of TRIC for noninvasive fetal genetic analysis.

## 5. Conclusions

In conclusion, we developed a modified TRIC workflow incorporating maternal immune-cell depletion to evaluate its effects on maternal-cell interference and trophoblast-associated characteristics. Among the evaluated strategies, CD56-mediated depletion demonstrated a more favorable profile for reducing maternal immune-cell interference and enhancing trophoblast-associated characteristics compared with CD45-mediated depletion. These findings suggest that CD56-based immune-cell depletion may contribute to the further methodological development of TRIC-based approaches for noninvasive fetal genetic analysis. Future studies integrating advanced molecular approaches, including single-cell sequencing and clinical genomic validation in larger cohorts, may further characterize trophoblast heterogeneity and determine the potential clinical applicability of cervical trophoblast-based testing.

## Figures and Tables

**Table 1:** Clinical characteristics and endocervical cell counts of the study participants.

Sample No.	Maternal Age	Fetal Sex	Gestational Age	Endocervical Cell
1	33	M	11 + 3	1.6 × 10^6^
2	37	F	7 + 5	8.8 × 10^5^
3	39	F	6 + 0	7.6 ×10^5^
4	35	-	7 + 2	1.7 × 10^6^
5	34	F	11 + 5	4.8 × 10^6^
6	33	F	10 + 1	9.6 × 10^5^
7	36	-	9 + 2	4.8 × 10^6^
8	34	F	16 + 3	2.1 × 10^6^
9	42	F	8 + 6	1.6 × 10^6^
10	37	M	15 + 1	3.0 × 10^6^
11	35	M	12 + 5	1.5 × 10^6^
12	36	F	14 + 0	1.8 × 10^6^
13	35	M	12 + 5	1.7 × 10^6^
14	37	M	12 + 0	1.3 × 10^6^
15	37	M	8 + 4	2.8 × 10^6^
16	37	M	12 + 4	4.0 × 10^6^
17	33	-	9 + 3	7.9 × 10^5^
18	37	M	15 + 1	8.0 × 10^5^
19	36	-	12 + 2	4.2 × 10^6^

**Table 2:** Antibodies used for immune cell depletion and trophoblast isolation.

Step	Antibody	Host	Surface Marker
1st capture	CD56	Mo	Natural killer (NK) cell
	CD45	Mo	White blood cell (WBC)
2nd capture	HLA-G (4H84 Clone)	Mo	Extravillous trophoblast (EVT)

**Table 3:** Primer sequences used for quantitative real-time PCR.

Gene	Sequence (5′ to 3′)	Tm (°C)	Size
HLA-G	GAGGCCAGTTCTCACACCCT	60	182 bp
	GCCTCACACTTGCGCTTGGA		
β-hCG	GAGATGTTCCAGGGGCTGCT	60	150 bp
	GGTGTTGACGGTGATGCACA		
Cytokeratin 7	GACGGAGTTGACAGAGCTGC	60	190 bp
	CTGGGCCTGGAGGGTCTCAA		
CD45	GACAGGGCAAAGCCCAACAC	60	150 bp
	TGGTTTGTGAGGGGCTCTCG		
CD56	GAGCTGGACAAAGGATGGGG	60	145 bp
	AGCCTTGTTCTCAGCAATGC		

**Table 4:** Summary of endocervical cell counts and HLA-G-positive cells according to immune cell depletion strategy.

Antibody Group *	Gestational Age	Endocervical Cell	Input Cell Number	HLA-G Positive Cell
NC (N = 16)	82.4 ± 18.5	3.7 × 10^6^ ± 4.0 × 10^6^	2.4 × 10^5^ ± 0.2 × 10^5^	6040.6 ± 5396.9
CD45 (N = 16)	82.4 ± 18.5	2.3 × 10^6^ ± 1.5 × 10^6^	2.4 × 10^5^ ± 0.2 × 10^5^	3937.5 ± 5357.6
CD56 (N = 19)	78.6 ± 20.2	2.2 × 10^6^ ± 1.4 × 10^6^	2.4 × 10^5^ ± 0.2 × 10^5^	5046.1 ± 6051.9

* Sample sizes for descriptive statistics were NC, *n* = 16; CD45-depleted, *n* = 16; and CD56-depleted, *n* = 19. The Friedman test was performed on the 16 complete matched specimens with data available for all three groups.

## Data Availability

The data supporting this study’s findings are not openly available due to sensitivity and are available from the authors upon reasonable request.

## Supplementary Materials

The following supporting information can be downloaded at https://www.mdpi.com/article/10.3390/diagnostics16172714/s1, Table S1: Participant-level allocation of cervical specimens and endocervical cells across experimental conditions.

## Abbreviations

The following abbreviations are used in this manuscript:

TRIC	Trophoblast retrieval and isolation from the cervix
NIPT	Non-invasive prenatal testing
cfDNA	cell-free DNA
CVS	chorionic villi sampling
uNK	uterine natural killer
CD45	cluster of differentiation 45
CD56	cluster of differentiation 56
NK	natural killer
HLA-G	Human leukocyte antigen G
HBSS	Hanks’ Balanced Salt Solution
DPBS	Dulbecco’s phosphate-buffered saline
Trypsin-EDTA	trypsin–ethylenediaminetetraacetic acid
PFA	paraformaldehyde
WBC	White blood cell
BSA	bovine serum albumin
cDNA	Complementary DNA
DAPI	performed with 4′,6-diamidino-2-phenylindole
SE	standard error
SD	standard deviation
ANOVA	analysis of variance
MACS	magnetic-activated cell sorting
CK7	Cytokeratin 7
β-hCG	β-human chorionic gonadotropin
CTB	cytotrophoblasts
STB	syncytiotrophoblasts
EVT	Extravillous trophoblast
WBCs	white blood cells
CD9	cluster of differentiation 9
