# Supplementary material for: Trophoblast Enrichment by Maternal Immune-Cell Depletion Using CD45 and CD56 Surface Markers in Trophoblast Retrieval and Isolation from the Cervix (TRIC)
Source: Diagnostics (Basel). 2026 Aug 25;16(17):2714. doi: 10.3390/diagnostics16172714 (PMC13565588; doi:10.3390/diagnostics16172714)
Supplement: Supplementary file 1 [file diagnostics-16-02714-s001.zip › Supplementary Table S1.pdf]

Supplementary Table S1. Participant-level allocation of cervical specimens and endocervical cells across experimental conditions.

Sample No.	GA	Endocervical cell	Input cell number		
			NC	CD45	CD56
1	7+2	$1.7x10^5$	$2.5x10^5$	$2.5x10^5$	$2.5x10^5$
2	11+5	$4.8x10^6$	$2.5x10^5$	$2.5x10^5$	$2.5x10^5$
3	10+1	$9.6x10^5$	$1.6x10^5$	$1.6x10^5$	$1.6x10^5$
4	9+2	$4.8x10^6$	$2.5x10^5$	$2.5x10^5$	$2.5x10^5$
5	16+3	$2.1x10^6$	$2.3X10^5$	$2.3x10^5$	$2.3x10^5$
6	8+6	$1.6x10^6$	$2.5x10^5$	$2.5x10^5$	$2.5x10^5$
7	15+1	$3.0x10^6$	$2.5x10^5$	$2.5x10^5$	$2.5x10^5$
8	11+3	$1.6x10^6$	-	-	$2.5x10^5$
9	15+1	$8.0x10^5$	$2.5x10^5$	$2.5x10^5$	$2.5x10^5$
10	14+0	$1.8x10^6$	$2.5x10^5$	$2.5x10^5$	$2.5x10^5$
11	12+5	$1.7x10^6$	$2.5x10^5$	$2.5x10^5$	$2.5x10^5$
12	7+5	$8.8x10^5$	-	-	$2.5x10^5$
13	6+0	$7.6x10^5$	-	-	$2.5x10^5$
14	12+5	$1.5x10^6$	$2.4x10^5$	$2.4x10^5$	$2.4x10^5$
15	12+0	$1.3x10^6$	$2.1x10^5$	$2.1x10^5$	$2.1x10^5$
16	8+4	$2.8x10^6$	$2.5x10^5$	$2.5x10^5$	$2.5x10^5$
17	12+4	$4.0x10^6$	$2.5x10^5$	$2.5x10^5$	$2.5x10^5$
18	9+3	$7.9X10^5$	$2.5x10^5$	$2.5x10^5$	$2.5x10^5$
19	12+2	$4.2x10^6$	$2.5x10^5$	$2.5x10^5$	$2.5x10^5$
